# Surgical Pathology Diagnostic Pitfalls of Hepatoblastoma

**DOI:** 10.1177/10668969211070178

**Published:** 2022-01-20

**Authors:** Finn Morgan Auld, Consolato M. Sergi

**Affiliations:** 1 6363 University of Ottawa, Ontario, Canada; 2 Anatomic Pathology Division, Children's Hospital of Eastern Ontario, Ottawa, ON, Canada

**Keywords:** Hepatoblastoma, pediatric liver tumor, hepatoblastoma diagnostic pitfalls, pediatric liver histology, hepatocellular neoplasm, beta-catenin

## Abstract

Hepatoblastoma (HB) is the most common malignancy within the rare cohort of pediatric primary liver tumors. It may arise sporadically or in association with germline mutations in specific genetic syndromes. Histogenesis recapitulates fetal hepatic development, however, this tumor can exhibit a markedly heterogeneous appearance both macroscopically and under the microscope. Histologic subtypes are classified based on morphologic appearance, with additional discrimination based on emerging molecular and immunohistochemical features. Numerous diagnostic pitfalls exist from clinical presentation through to ancillary testing; at all stages, the surgical pathologist must be discerning and open to collaboration with colleagues of different specialties. Problematic areas include the adequacy of tissue sampling, correlation of histology with radiologic appearance and alpha feto-protein (AFP) serology, forming a diagnostic consensus within the pediatric pathology community and choosing a shrewd immunohistochemical panel. This review discusses the sequence of events leading up to histologic assessment, and the nuances of microscopic evaluation. Along the way, pitfalls are highlighted, providing a tool for the surgical pathologists to support their individual approach.

## Introduction

Hepatoblastoma (HB) is the most common primary liver tumor of pediatric populations; while sporadic cases in adults and older children have been reported, the overwhelming majority occur in children younger than three years.^1^ The incidence of HB appears to be increasing. Epidemiological data are limited, however a steady increase of 4.3% per year between 1992 and 2004 has been documented in children younger than 19 years.^2^ This trend seems to continue into recent literature. Feng *et al* illustrate an annual percentage increase of 2.2% between 2000 and 2015.^2^ Various explanations have been proposed: cases may be revealed by screening young children with the genetic propensity to develop HB, using abdominal ultrasound and alpha feto-protein (AFP) serology;^3,4^ another likely contributor is the tremendous advance in neonatal medicine, improving the survival of infants who bear dual risk factors for HB development, prematurity and very low birth weight of <1500g.^1^

A tumor of embryonal origin, HB, recapitulates the hepatic development of fetal life.^5^ Its striking heterogeneity can be explained by its origin, arising from the aberrant division of both primary hepatoblasts and human fetal liver multipotent progenitor cells. Hepatoblasts are hepatic stem cells responsible for sequenced progression from fetal to fully differentiated adult liver tissue. Liver multipotent progenitor cells are poorly differentiated at the outset and have the potential to differentiate into a variety of tissue types, from those native to the liver, to fat and bone.^1^ Distinct morphologic subtypes exist, classified according to their tissue components. Further categorization is described according to molecular features, as advances in immunohistochemical techniques allow the discrimination of morphologically similar tumors.^6^ While the majority arise sporadically, up to a third of HB cases are associated with a genetic syndrome or congenital anomaly.^7^ Among those most often implicated are Beckwith-Wiedemann syndrome, familial adenomatous polyposis, Edward syndrome (trisomy 18) and Down syndrome (trisomy 21).^7–9^ In synchrony with research teasing out the histologic subtypes, attention is paid to the unique gene expression profile of tumors, which may be helpful in stratifying their behavior. In particular, the beta-catenin pathway is strongly implicated the genesis and progression of HB, and thus represents a tantalizing therapeutic target.^10,11^

In order to procure an accurate and timely diagnosis for this young patient population, it is important to be aware of common diagnostic pitfalls. This review discusses histopathological diagnosis, particularly since case numbers in most departments are too low to form a reliable internal database for comparison. The interface between clinical medicine and histology is also explored, as each relies upon the other to influence therapeutic decisions.

## Clinical Presentation

Patients with HB most commonly present with abdominal distention or a palpable mass in the right upper quadrant. A vague constellation of symptoms is often associated, including weight loss, anorexia, nausea, vomiting, and diffuse abdominal pain. Jaundice may present in a slim minority, approaching 5% resulting from biliary impingement by the tumor mass.^1,12^ Rarely, precocious puberty or frank virilization may prompt investigation due to ectopic sex hormone of beta hCG production by tumor cells. Laboratory workup reveals anaemia in the majority (70%) and thrombocytosis in approximately 50%, thought to be due to thrombopoietin production by tumor cells.^13^ An overwhelming majority of patients have raised serum AFP at presentation: while helpful as an adjunct to clinical suspicion, AFP levels are physiologically raised at birth and may not normalize for the first 6 months of life.^7,12^ Additionally, AFP levels may not be raised in tumors comprising poorly differentiated cells, such as the small cell undifferentiated histologic subtype.^1,7,14^ However, an awareness of the limitations of investigative modalities prevents an over-reliance on individual parameters or laboratory values. At all times, it is prescient to consider differential diagnoses when evaluating the clinical history. Liver tumors are a diverse group of neoplasms with radically different management protocols and prognoses. In the context of a large, aggressive hepatocellular tumor with high serum AFP levels, a newer entity should be born in mind: *hepatocellular malignant neoplasm, not otherwise specified (HCN-NOS)* is a subset of tumors described at the 2014 COG liver tumors symposium.^15^ The discussion surrounding this consensus classification is explored further in the text, however an awareness of the spectrum of differential diagnoses is vital before slide review.

## Radiologic Assessment

Radiologic imaging is essential for identification of tumors, pre-operative planning and monitoring for recurrence. Congenital HB may be detected as early as the start of the third trimester by maternal ultrasound, presenting as a single, solid, echogenic lesion.^9,13^ In children, CT and MRI scan aid in distinguishing HB from other tumors of childhood, such as mesenchymal hamartoma and pediatric hepatocellular carcinoma (HCC), though in this context MRI is preferred due to its superior discernment of mixed soft tissues.^1,5,16^ MRI may reveal an epithelial tumor component, vascular invasion and the presence of fibrotic bands. Although considered a soft sign, intra-tumoral calcifications on CT add weight to a differential diagnosis of HB, since these are present in approximately 50%.^5^ Ultrasound is clinically easier to perform in the pediatric population since anaesthesia is not required; a tumor mass and its internal components can be mapped using ultrasound, however, this modality lacks the refined image quality of MRI.^17^ To monitor recurrence post-treatment, Fluorodeoxyglucose positron emission tomography (FDG-PET) can be used, particularly to identify early recurrence.^1,5^ Unfortunately, due to the low FDG avidity in cells of pure fetal type, these tumors may be challenging to distinguish from normal background liver, and recurrence may not be readily identified. In such circumstances, imaging should be taken in the clinical context of serum AFP levels, which fall post-treatment and tend to rise with recurrence and repetitive CT surveillance.^3,13,18^

## Diagnostic Considerations

The gold standard of diagnosis remains histological examination of the tumor mass, supported by clinical, biochemical and radiological assessment.^1,19–21^ As preoperative chemotherapy is increasingly becoming the standard of care, core needle biopsy and fine-needle aspiration are often utilized in the first instance, to provide tissue for paraffin preparation and immunohistochemistry.^5,13^

Fine needle aspiration (FNA) is rarely utilized in the diagnostic approach to liver lesions. However, it may be used alongside radiology when a benign lesion is suspected. It is a tempting modality due to its less invasive sampling method. However, it is not generally regarded as providing sufficient evidence for diagnosis, mainly when dealing with a mixed or undifferentiated pattern.^19,21^ The College of American Pathologists emphasizes the difficulty in distinguishing well-differentiated hepatocellular malignancy from regenerative changes and benign proliferations.^19,21^ FNA has been described in the diagnostic workup of mesenchymal hamartoma: HB is a major differential for the slightly less common mesenchymal hamartoma and has its own distinctive cytological features, such as clusters of tumor cells showing acinar and trabecular patterns, small round tumor cells with a high nuclear to cytoplasmic ratio, hyperchromatic nuclei with prominent nucleoli, and the presence of extramedullary haemopoiesis.^17,22^ Due to the heterologous nature of both HB and mesenchymal hamartoma, and the narrow sampling potential of FNA, these two lesions with vastly different clinical trajectories can be mistaken for one another. FNA may miss the spindle cell mesenchymal element classically associated with mesenchymal hamartoma. In addition, mesenchymal hamartoma may be associated with increased serum AFP, further obfuscating the clinical distinction.^20,22,23^ Taking this into account, FNA is not considered adequate for diagnosis of HB. The current recommendation for diagnosis of HB is biopsy if surgical resection in the first instance is not an option. Examples of lesions not amenable to primary resection include large vessel invasion or those that are multifocal.^1,5,16^ The former will warrant pre-surgical chemotherapy and the latter may remain unresectable.^13,15^ Needle biopsy optimally results in 4 to 6 liver cores, however, more is always considered better.^19^ The biopsy may take place percutaneously or via a more invasive laparoscopy or laparotomy, guided by characteristics of the tumor such as vascularity which make it inaccessible to less invasive biopsy methods. Notably, a diagnosis of pure well-differentiated fetal type HB can only be made on a resection specimen, where all tumor cells are seen to be of distinctive fetal morphology, not on biopsy alone or following chemotherapy.^5,13,19^ This safeguards against the omission of a smaller, less differentiated subtype rendering the sample a mixed tumor with a different clinical path.

## Genetic Associations

In contrast to the stepwise accumulation of somatic mutations often implicated in adult malignancies, pediatric tumors appear to develop more abruptly, pointing to highly penetrant somatic mutations in undifferentiated cell lines or genetic predisposition. The latter has been described in association with germline mutations in specific genetic syndromes, which account for approximately 15% of HB cases.^8,24,25^

Myriad cytogenetic changes have been identified in HB; those failing to maintain normal chromosome number appear to be key to tumor development. In addition, germline and somatic mutations compound developmental factors, such as low birth weight and prematurity, to influence HB induction and growth.^1,6,8,26,27^

Mutations implicated in the development of HB often involve the Wnt signaling pathway, more specifically, relating to the beta-catenin protein, a transcriptional cofactor.^10,11,28^ Wnt signaling is intrinsic to embryonic development, promoting organized cellular proliferation and differentiation.^10,29^ It is fitting that the most frequently reported mutation in HB is in *CTNNB1*, a gene encoding the beta-catenin protein, playing an important role in cell communication and adhesion.^6,8,26^ *AXIN1* and *AXIN2*, downstream components of the Wnt pathway, contribute to beta-catenin degradation.^10,28^ Zhang *et al* (2019) describe that all beta-catenin mutations are tumorigenic when co-expressed with mutant *YAP*; they also provide context for the striking biological diversity of HB, by describing the influence of myriad beta-catenin mutation identities on tumor characteristics such as growth rate, metabolic profiles, and diverse histological appearance.^10,30^ N6-Methyladenosine (m6A) is a modification of RNA with rampant involvement in normal cellular processing and malignant disease alike. It has been implicated in the development of a variety of cancers via its governance of gene expression. Genes associated with m6A formation and function feature prominently in HB, underscoring their role in its promotion and clinical course.^28,31^ Such genes include *METTL3*, which consequently influences *CTNNB1* as a downstream target, alongside its heterodimer partner *METTL14*.^6,26,28^ While the sea of genetic aberrations associated with HB can be overwhelming to behold, the pathologist is urged to remain aware of new discoveries, which are frequent. The focus of research is often the prediction of tumor behavior, tumor response to current therapies and identification of novel therapeutic targets, all of which have a tangible impact on patients. One example from recent literature is the described relationship between METTL3 expression and the clinicopathological characteristics of patients with HB. Increased METTL3 expression was associated with frequent recurrence and poor prognosis.^31^ Such developments serve to better stratify patient risk and inform management decisions.

Several case reports describe the co-occurrence of HB with other developmental disorders, such as Hirschsprung disease. In this context, it is noted that Hirschsprung disease may be associated with syndromes predisposing towards cancer.^32,33^ Regarding a shared genetic aetiology, the *RET* proto-oncogene is a likely culprit. Pathogenic mutation of *RET* is the most common genetic aberrance identified in patients with Hirschsprung disease and has been extensively studied, implicating over 100 unique mutations of various types.^33^ *RET* exerts its effects through a variety of pathways some of which, like PI3/AKT, have rarely been described as driving HB.^33^ Pathogenic mutations of *RET* are diffusely involved in a number of solid organ malignancies and cancer syndromes, by virtue of the role of *RET* as a mediator of cell proliferation, survival, migration, apoptosis and differentiation.^12,33^

## Bringing a Consensus

A number of collaborative efforts internationally has elaborated correlation between tumor biology, clinical decision making, and patient outcome; expansion of the Pediatric Liver Tumors Consensus Classification (PLTCC) incorporated histopathology into the Children's Oncology Group (COG) protocols in order to stratify risk and treatment decisions.^15^ The rarity of pediatric liver cancers has consistently limited large biological studies. Interobserver variability is commonplace, even among the world's experts. Within the consensus classification, a significant sticking point involved identifying the presence of a minor (<50%) small cell component, or the coexistence of HB with pediatric HCC.^15^ In establishing the diagnosis of such a rare malignancy with the security it is due, consultation for expert second opinion is consistently recommended.^15,19,34,35^

Within the cohort of pediatric liver tumors, it is imperative to distinguish not only between HB subtypes, but between different entities which may present similarly to clinicians and pathologists alike. Pediatric HCC is a small but significant contribution to this cohort, making up approximately 20% of malignant pediatric liver tumors.^5,15^ While pediatric HCC tends to present in an older age group, there is significant morphologic overlap with HB.^36^ A misdiagnosis is critical, as chemotherapeutic regimens utilized with success in HB have no impact on the activity of pediatric HCC.^15^ Like HB, progress in the field of bespoke pediatric HCC management has been limited by the heterogeneity of the tumors and the paucity of cases and samples for study.^36,37^

Within the consensus classification, a group of tumors resisted further classification, demonstrating a variety of morphologies. Some cases exhibited features of both pediatric HCC and HB within the same tumor.^15^ This constellation of appearances which preclude exact classification has been termed *hepatocellular malignant neoplasm, not otherwise specified (HCN-NOS)*.^15^ Prior to description of this entity, a series of seven highly aggressive and chemo-resistant epithelial liver tumors was inducted into the literature by Prokurat *et al* Similar to cases assessed in formation of the consensus classification, Prokurat *et al* noticed a mixture of poorly differentiated cells, HCC-like cells and HB-like cells at varying stages of differentiation, often occurring within the same tumor mass.^15,38^ As a result of these observations, this recalcitrant group of neoplasms was termed *transitional cell tumors of the liver*.^38^ While such tumors may represent a transitional lineage with a common precursor, there are myriad other possibilities such as chemotherapy-induced change to previously identified tumors, or a novel neoplasm of true transitional cell origin.^15,36^ As such, the consensus classification removed the potentially obfuscating title of *transitional cell tumor* in favor of HCN-NOS.^15^

HB is so named for its histological appearance, chiefly the hepatic epithelial tissue resembling immature fetal or embryonal liver.^5^ Tumor cells exhibit a variety of appearances, from primitive blastema through poorly differentiated embryonal hepatocytes to fetal hepatocytes. Regarding histological assessment, the variability of cytology and tumor architecture, both within and between cases, is a potential pitfall even for an experienced pathologist.^37^ The rarity of HB means that even larger centers do not receive these specimens with a frequency necessary to develop an internal database of experience.^5,15^ To combat this, a systematic central histopathological review of pediatric liver tumors is encouraged, based on the work of cooperative groups such as COG. The subsequent international PLTCC developed a classification system based on a cohort of 50 pediatric liver tumor cases, which stratified treatment decisions and served patient interests based on internationally shared experience.^1,15^ The description or rare and novel histologic subtypes, such as the presence or absence of teratoid features within the mixed epithelial and mesenchymal subtype, provided a much-needed framework for diagnosticians worldwide. In addition to the interpretation of heterogeneous histology, accurate and timely diagnosis of HB was challenged by the lack of immunohistochemical stains, lack of or insufficient clinical history, and sampling limitations in the form of small or limited biopsies.^1,15^ In this regard, the pathologist relies upon the expertise of her clinical colleagues to provide sufficient primary and ancillary diagnostic material.^12,15^ Considering these challenges of rarity and consensus, HBs have certain identifying qualities macroscopically and microscopically, which will be discussed here.

## Macroscopic Appearance

Recapitulating radiological findings, HBs are commonly seen grossly as a large, solitary mass (80%) localized to the right lobe of the liver in the majority (60%).^13,18^ Macroscopic extension into the vena cava identified in over half.^19^ Even at this early stage, the pathologist should be mindful of differential diagnoses. If the tumor is seen to arise in the left lobe of the liver, an uncommon behavior for the majority of primary liver tumors, the fibrolamellar variant of pediatric HCC should come to mind.^13^ This tumor is an important consideration as it portends a poor prognosis, and otherwise closely resembles the appearance of HB macroscopically.^13^ On sectioning, the cut surface of a HB has a bulging appearance. The lesion may be partially encapsulated and involve cystic change.^19^ Areas of necrosis and haemorrhage are frequently observed. The color of the tumor surface varies depending on its histologic subtype: the better-differentiated fetal HB is tan-brown in color, owing to its similarity to the normal liver. Other subtypes and mixed subtypes are more variegated, ranging from pale-tan to white to green.^1,19^

**Figure 1. fig1-10668969211070178:**
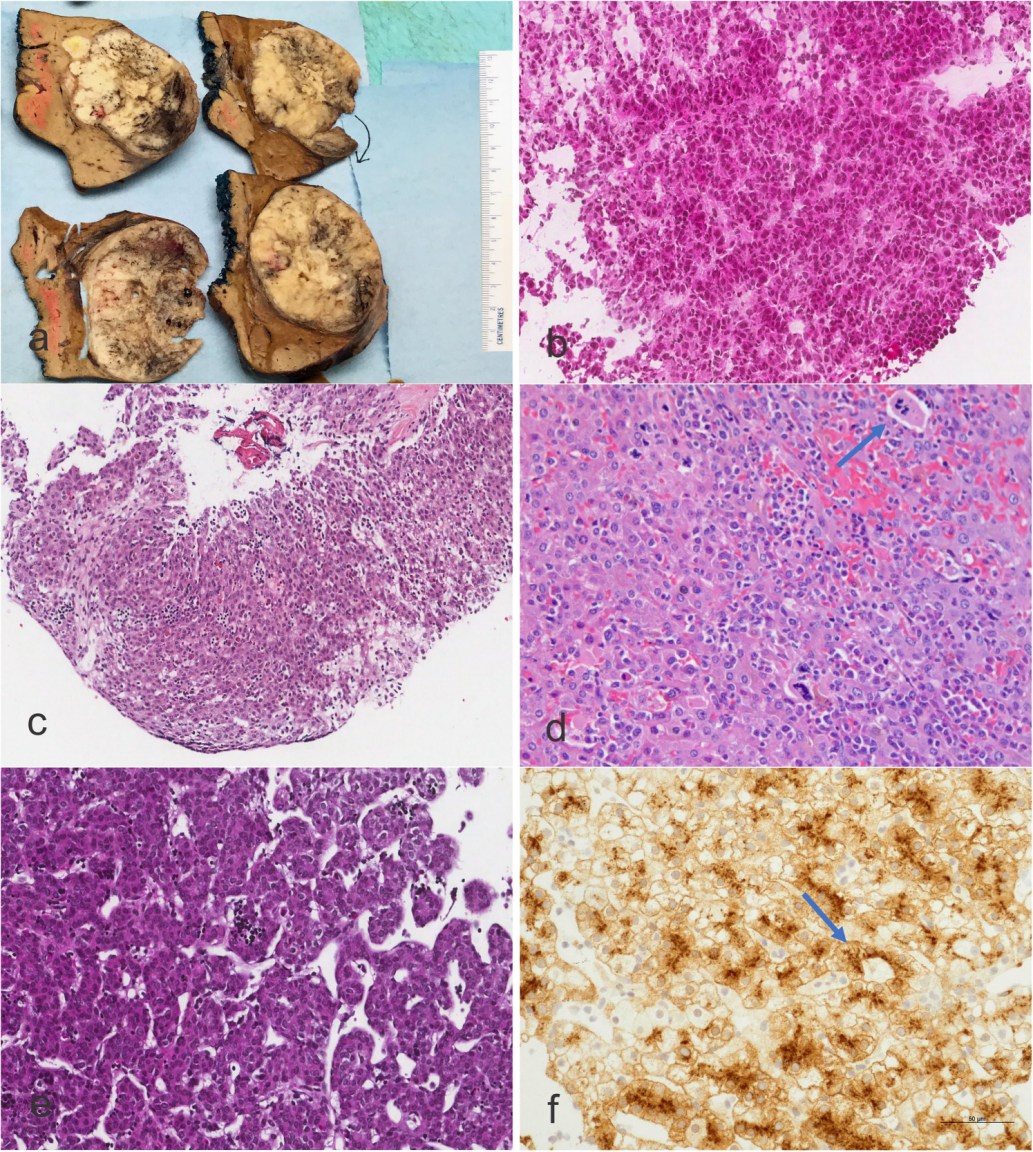
a) Gross picture of a hepatoblastoma extensively invading the liver and perforating the glisson's capsule. The tumor appears solid, grey but also displays some hemorrhage. b) Microphotograph of an intraoperative frozen section performed during a liver biopsy to identify the viability of tissue for COG studies and differentiation from hepatocellular carcinoma or embryonal sarcoma. The presence of a trabecular arrangement of the tumor cells displaying hepatocytic morphology is quite typical. There is a resemblance to the fetal liver (Hematoxylin-eosin staining, X100, original magnification). c) Microphotograph of a fetal hepatoblastoma with numerous hematopoietic cells between the trabecular arrangement of the hepatoblasts (Hematoxylin-eosin staining, X50, original magnification). d) Microphotograph of an embryonal hepatoblastoma with an increase of the nucleus to cytoplasm ratio and focal megakaryoblasts (arrow). Megakaryoblasts need to be confirmed using anti-platelet immunohistochemical markers (eg, CD61). Megakaryoblasts may be mis-interpreted as marked atypia or anaplasia in a setting of a small round blue cell tumor. (Hematoxylin-eosin staining, X200, original magnification). e) Microphotograph of an embryonal hepatoblastoma showing trabecular arrangement in cords and scattered islands of extramedullary hematopoiesis (Hematoxylin-eosin staining, X200, original magnification). f) Microphotograph of a fetal hepatoblastoma showing glypican 3 staining. The arrow shows a periluminal accentuation of the staining. (Anti-Glypican 3 immunostaining, Avidin-Biotin Complex, X200, original magnification).

During gross examination of a resection specimen, margins are routinely inked and the specimen is photographed and mapped (Figure 1a). Keeping a map to hand will assist with histologic interpretation, allowing correlation between the microscopic and macroscopic appearance, which is often heterogeneous. Block sampling of one section per centimeter of tumor is considered appropriate, paying special attention to macroscopically diverse areas.^19^ This ensures that the tumor is properly subclassified, and smaller mixed areas are not omitted. This becomes especially important when unfavorable histopathologic features, such as a small undifferentiated component.^5,14^ When making the first cut in the block selection process, the vascular margin should be submitted first, to prevent carry-over of tumor and an artificially positive margin.^5^

## Microscopic Features

HB is comprised of both epithelial and stromal components: the former can be either embryonal or fetal and the latter may exhibit a mixture of connective tissue and heterologous elements such as cartilage, bone and skeletal muscle.^24,25^ Arrest or hijack of the differentiation pathway of liver multipotent progenitor cells and primary hepatoblasts explains the markedly heterologous potential of HB.^7,39^

**Figure 2. fig2-10668969211070178:**
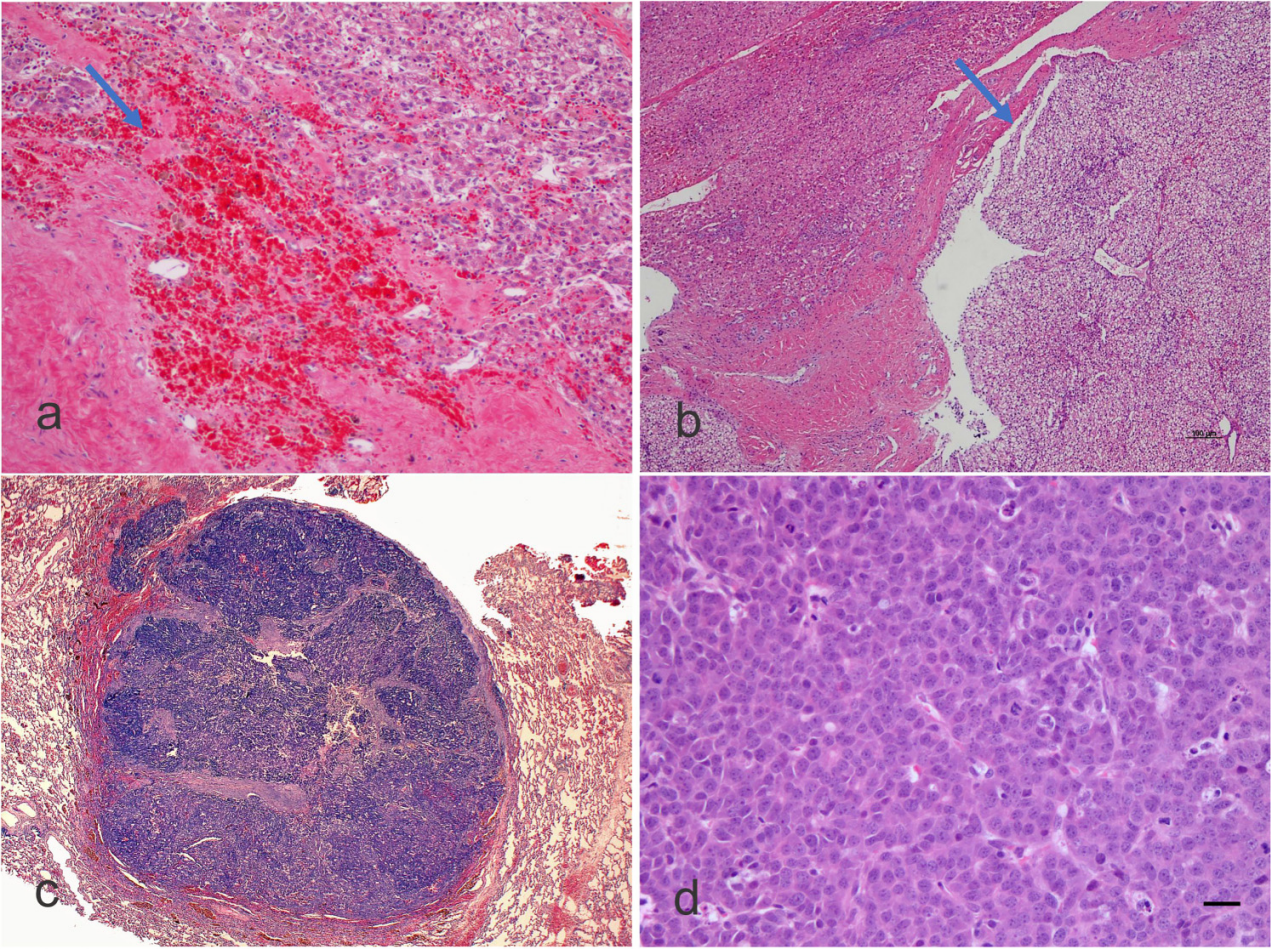
a) Microphotograph of a hepatoblastoma following chemotherapy with degenerations including fibrosis and hemorrhage (arrow) and some residual hepatoblasts showing some signs of maturation with clear cytoplasm (hematoxylin-eosin staining, X100, original magnification). b) Microphotograph of a hepatoblastoma following chemotherapy and clear cell change (arrow), which needs to be taken into the differential diagnosis with other tumors potentially showing clear cell morphology. Some islands of hematopoiesis could be recognized (Hematoxylin-eosin staining, X40, original magnification). c-d) Microphotograph showing lung tissue of a partial pneumectomy including a metastasis of a hepatoblastoma of embryonal type. The high magnification in d) shows a packed arrangement of the cells (c: hematoxylin-eosin staining, X20, original magnification; d: hematoxylin-eosin staining, X200, original magnification).

HB is classified according to histologic type, and the ratio of tissue types within the same tumor; broadly, the two major categories are epithelial and mesenchymal. Four main subtypes are under the epithelial umbrella: fetal, embryonal, small cell and macrotrabecular.^13,15,40^ Mixed subtypes occur, the most common of which is mixed fetal/embryonal. The presence of a mesenchymal component in addition to an epithelial component of any subtype defined another mixed pattern, a mixed epithelial/mesenchymal tumor.^12^. Within the mixed epithelial/mesenchymal subtype, a further division is described, based on the presence or absence of heterologous elements representative of all germ layers. These minor subtypes are named teratoid and non-teratoid, respectively (Figure 1 b–f; Figure 2).^12^

Other rare variants have been described in the literature outside of these established categories, often as case reports. For example, neoplastic cells may differentiate along a pathway reminiscent of cholangiocytes, resulting in bile duct-like structures within the tumor mass.^12,18,27^ This is particularly problematic following pre-operative chemotherapy, which can induce a ductular reaction in the periphery of the tumor.^12^ In slightly older children of over 5 years, tumors may exhibit intermediate features between hepatoblasts and hepatocytes. Such tumors are termed transitional liver cell tumors and do not respond to conventional HB therapy.^5^

There is no relationship between the child's age and the observed subtypes of HB, which tend to be randomly intermingled.^19^ As patient age increased from infancy to beyond 5 years, however, the differential diagnosis of pediatric HCC should be seriously considered, as incidence approaches and overtakes that of HB.^5^ The main distinguishing feature between these two tumors is the unique stromal component assigned to HB, however a significant diagnostic challenge is posed by the overtly HCC-like macrotrabecular variant.^5,12,13^

The major subtypes appear as follows:

### I. Fetal Subtype

Neoplastic cells are small, uniform, and polygonal in shape, growing in sheets or as thinner trabeculae, 1 to 3 cells thick. Cells have distinct borders and are more cytologically bland than their less well-differentiated counterparts, with generally inconspicuous nucleoli and clear to finely granular cytoplasm.^1,5,12^ When scanning a slide on low power, a characteristic “light and dark” pattern emerges, caused by a varying amount of glycogen and lipid within the tumor cells. Mitotic activity is usually rare. However, a subset of mitotically active fetal HB exists, the threshold for which is >2 mitoses per 10hpf.^1,5^ The fetal subtype is the most well-differentiated, recapitulating the appearance of fetal hepatoblasts.^12^

### II. Embryonal Subtype

Morphologically, tumor cells correspond to the embryonic stage of liver development. The cells are more primitive in appearance than those of fetal HB, exhibiting high NC ratios, scanty cytoplasm, and angulated nuclei.^5^ Cells may form rosettes, show brisk mitotic activity and readily undergo necrosis. Rosetting may at a glance look like primitive bile ducts or glandular structures; care should be taken to view these in the context of the wider histology and macroscopy.^12^ A purely embryonal tumor is rare, and a main differential consideration is blastemal tumors such as a Wilms tumor. Most embryonal tumors, when extensively sampled, contain foci of fetal type morphology.^1,5,7^

### III. Mixed Fetal and Embryonal Subtype

This subtype contains both fetal type epithelium and primitive embryonal cells in varying proportions, forming sheets, ribbons, trabeculae, or clusters.^5,12^ Cell borders are less well defined than in purely fetal HB, and pleomorphism is milder than the pure embryonal subtype, apart from in areas where this morphology dominates.^12^

### IV. Macrotrabecular Subtype

This rare morphological type is defined by the presence of a repetitive pattern of trabeculae, frequently at least 10 to 20 cells thick.^5,19^ The epithelium forming trabeculae can vary in its type, from pure fetal to embryonal, to a third hepatocyte-like cell type. The latter closely resembles pediatric HCC and provides a significant diagnostic challenge at microscopy.^5^ To differentiate macrotrabecular HB from pediatric HCC or HCN-NOS, close attention is paid to the staining pattern of beta catenin: macrotrabecular HB exhibits nuclear staining in line with other classic HB subtypes, pediatric HCC meanwhile shows membranous staining, and often has thicker trabeculae.^5,37^ Macrotrabecular change can be present focally in other subtypes, in which case the tumor is classified according to its dominant portion.^12^

### V. Small Cell Undifferentiated

This subtype comprises sheets of small, discohesive cells of non-specific morphology.^5,14^ While an entire biopsy specimen may be comprised of such cells by chance, a far more common scenario is nests of small cells with pale nuclei interspersed within an epithelial HB showing mixed fetal and embryonal components.^21^ In cases where small cells appear to dominate, they must be differentiated from other “small round blue cell tumors’ such as Wilms tumor, neuroblastoma, Ewing sarcoma, rhabdomyosarcoma, desmoplastic small round cell tumor and lymphoma. In particular, malignant rhabdoid tumors must be separated from the small cell component of HB, since their diagnosis confers a poor prognosis and requires an entirely distinct management protocol. Histologically, malignant rhabdoid tumors may be distinguished by a characteristic appearance, comprising tumor cells with eccentric, pink cytoplasmic inclusions and vesicular nuclei. Malignant rhabdoid tumors show global loss of INI1 staining due to *INI1* gene mutation, whereas at most, INI1 loss is focal in HB and confined to the small cell undifferentiated component.^5,18,21,41^

### VI. Mixed Epithelial and Mesenchymal Subtype

This pattern is defined by the presence of any mesenchymal element in addition to the epithelial component. The mesenchymal element is usually osteoid, however it may take the form of cartilage, muscle, fat and primitive spindle cell mesenchyme.^12^ The mesenchymal element shows a similar staining pattern to its epithelial counterpart, with positivity for beta catenin in a nuclear distribution as well as keratin proteins.^1,5,40^ In approximately 20% of these mixed tumors, heterologous elements representing all germ cell layers are present, provoking further description as *mixed epithelial and mesenchymal subtype with teratoid features*.^12^ The teratoid component comprises primitive neuroectodermal structures such as glia or melanocytes, admixed with components showing endodermal differentiation such as gut.^12^

## The Histopathology Report

Appreciating the close relationship between clinical investigation and histopathology, the standardized report emphasizes both. This pronounces the need for excellent communication between the pathologist and their clinical team. It is good practice to, where possible, delve into the clinical record in order to appreciate the context of a histology slide. In assessing HB, the pathologist must be diligent in their understanding of the patient journey prior to diagnosis and in the perioperative period. The report contents is displayed in Table 1.^19^

**Table 1.** Contents of the Histopathology Report.^18.^

Parameter	Comments
Procedure	Biopsy versus resection
Tumor site	
Tumor size	Each nodule
Tumor focality	
Macroscopic extent	
Preoperative treatment	
Histologic subtype	%, differentiation and mitotic activity
Treatment effect	
Margin status	
Capsular surface involvement	
Lymph-Vascular invasion	Macroscopic versus microscopic
Regional lymph node status	
COG staging	
Additional pathologic findings	Cirrhosis, iron overload, hepatitis
Serum AFP level	<100ng/mL versus ≥100ng/mL
Ancillary studies	INI1 expression: retained or lost, beta-catenin, glypican 3, other

Ancillary studies, including immunohistochemistry, are vital in facilitating accurate diagnoses, differentiating between the diverse cohort of hepatocellular tumors and normal immature liver tissue. The routine immunostains recommended to support HB diagnosis are: beta catenin, glypican 3, glutamine synthetase, and INI1.^21^ Since HB recapitulates the differentiation of fetal hepatocytes and their progenitors, staining patterns can differ significantly between subtypes. Staining is also variable within tumors of the same morphologic type, a nod to their internal heterogeneity and complex biochemical behavior. The immunohistochemical description of HB subtypes is a rapidly evolving area of research, particularly as the genetic basis of protein expression by tumor cells is progressively understood. The following represents an approach to the standard immunohistochemistry panel, with some honorable mentions to assist with the diagnosis of rarer variants.

### I. HB versus Normal Liver

Glypican 3 has a distinctive staining pattern in well-differentiated fetal HB, with fine pericanalicular positivity.^5,19^ Mitotically active fetal HB and embryonal components have a coarser cytoplasmic pattern. Small cell undifferentiated, mesenchymal elements and rarer subtypes such as cholangioblastic, are uniformly glypican 3 negative, as is normal liver tissue.^19^ In addition to glypican 3, beta-catenin and glutamine synthetase are useful adjuncts to differentiate HB from normal liver. Nuclear beta-catenin and diffuse glutamine synthetase staining, albeit weaker in intensity than glypican 3, is a hallmark feature of tumor cells, specifically those of epithelial subtypes.^5,19^ As tumors move along the differentiation pathway, the staining pattern necessarily changes. Of particular importance, beta-catenin can shift from the hallmark strong nuclear positivity to a more membranous pattern.^11^ An additional marker utilized is CD34: diffuse capillarization is noted as a unique feature of HB, illuminated by this pattern of CD34 staining and differentiating it from benign lesions and background liver. This appearance indicates that sinusoids within the tumor are lined by endothelial cells.^12,18^

### II. HB versus Pediatric HCC

There is currently no immunostain or panel to differentiate HB from pediatric HCC unequivocally. Beta-catenin is generally less intense in a nuclear distribution in pediatric HCC when compared with HB, however, a rarer subset is strongly and problematically positive.^5,19^ The balance is tipped towards HB in the presence of glutamine synthetase and cyclin D1 positivity, and certain genetic markers are more commonly encountered, such as chromosome 1p abnormalities and trisomy of chromosomes 2, 20, and 8.^5,19,25,27^ However, as with immunohistochemical staining, this distinction is not definitive, and the same genetic markers are highlighted in up to 40% of pediatric HCC.^5^

### III. Evaluation of the Embryonal Component

Beta-catenin is a reliable but non-specific marker of the embryonal subtype, showing strong nuclear positivity.^5,10,29^ Since embryonal HB represents a less mature tumor than well-differentiated fetal HB, staining with markers of later development may not be as uniform. Glypican 3 ranges from strong to absent, and glutamine synthetase is equally as variable, ranging from patchy single or scattered cells to negative.^19^

### IV. Evaluation of the Small-Cell Undifferentiated Component

A major differential for a small cell undifferentiated component is a malignant rhabdoid tumor. As such, assessment with INI1 is critical.^13,19,40^ Global loss of INI1 staining in the presence of rhabdoid morphology indicates a malignant rhabdoid tumor, whereas patchy loss, preservation and in some cases increased density of INI1 in tumor cells within the small cell undifferentiated component, supports the diagnosis of HB.^12,14,19,21^ Beta-catenin usually stains the small cell undifferentiated component in the same positive nuclear distribution as other HB subtypes, however glypican 3 and glutamine synthetase are often negative; this subtype may also stain positive for vimentin and keratin proteins.^12,14,19^

### V. Immunohistochemical Behaviour

It is currently not possible to consistently predict which patients will respond to which treatments.^13,29^ This invites a role for immunohistochemical stains beyond diagnostics, into the prediction of tumor behavior. For example, investigation into immunophenotypic differences between chemoresponsive and non chemoresponsive tumors highlight a loss of c-myc and diffuse expression of CK19 and Survivin in aggressive, less chemoresponsive tumors, with the reverse staining pattern in tumors which responded well to chemotherapy.^29^ Keratin-19 or cytokeratin-19 is a 40 kDa type I cytoskeletal protein that in humans is encoded by the *KRT19* gene. Given the explicit implication of Wnt/beta-catenin signaling in HB development, studies have attempted to evaluate this pathway as a potential therapeutic target.^11^ Activation of Wnt/beta-catenin signaling regulates hallmarks of tumor progression such as differentiation, metastases and invasive potential. Therefore, suppression of this system can potentially attenuate the progression and development of HB.^11,30^ Table 2 details the immunohistochemical markers expressed in the single subtypes of HB.

**Table 2.** Comparison of Staining Patterns in HB Subtypes and Common Pitfalls.

	Glypican 3	Beta-catenin	Glutamine synthetase	INI1
Normal Liver	-	-	-	+
Well-differentiated fetal HB	+ fine pericanalicular	+ / variable	+	+
Mitotically active fetal HB	+ cytoplasmic	+ / variable	+	+
Embryonal HB	+ / variable	+ / variable	+ / variable	+
Small cell undifferentiated HB	-	+ nuclear	- / +	+ / patchy / increased
Minor HB subtypes: cholangioblastic, mesenchymal, teratoid	- / + variable (teratoid)	+ / variable	-	+
HCC	+ / variable	+ / variable	+ / variable	+
Malignant rhabdoid tumor	+ / - variable	- / + rare	+ / variable	-

## Common Pitfalls for the Histopathologist

By distilling the sequence of investigation and diagnosis relating to HB, certain common pitfalls come to light. The following potential trips are of particular importance to the surgical pathologist, who must synthesize abundant clinical, histological and ancillary information prior to reaching a conclusion:

1. *Is the tissue adequately sampled?*
  - FNA is not considered to provide sufficient evidence for diagnosis.
  - Biopsy is considered diagnostic, but even multiple cores may fail to capture heterologous elements in a mixed tumor.
  - Resection is optimal but not always possible, specifically in multifocal tumors or those requiring pre-operative chemotherapy.
  - Read the operation note if it is available: consider whether the tumor has been diffusely sampled by core biopsy whether there have been intraoperative challenges, and whether intraoperative challenges have impacted the type of biopsy performed.
2. *How much emphasis is placed upon histologic subtype?*
  - Various features of the tumor impact prognosis, in addition to the histologic subtype. Such features are captured by reporting proforma and include: tumor size, focality, margin status, COG stage, metastatic spread and response to treatment.
  - There is no prognostic significance of identifying mixed morphology.
  - Pure well-differentiated fetal morphology is considered prognostically favorable, as these tumors may be managed with surgery alone.
  - Small cell undifferentiated morphology comprising >75% of the tumor mass is deemed prognostically unfavorable, as such tumors respond less well to current treatment modalities.
  - The prognostic significance of a small cell undifferentiated component as a smaller proportion of the tumor mass is undetermined.
3. *How has the diagnosis of well-differentiated fetal HB been established?*
  - Well-differentiated fetal HB can only be diagnosed in a resection specimen, where all tissue is examined.
  - The younger the patient, the more challenging it is to distinguish well-differentiated fetal HB from normal infant liver: fetal tumor cells are larger with a greater NC ratio, however this is problematic on core biopsy where there may not be a good source of internal control for the eye of the observer.
  - The establishment of pure well-differentiated fetal HB is clinically relevant as treatment may involve surgery alone.
4. *Does the imaging support your histopathological assessment?*
  - Radiological correlation is paramount, particularly when subtyping a mixed tumor. Heterologous mesenchymal elements such as bone are readily observed on imaging and may be neglected by core biopsy.
  - Imaging may fail to differentiate between mesenchymal hamartoma and HB in cases where mesenchymal hamartoma is vascular, complex or exhibits features usually associated with malignancy such as necrosis. Mesenchymal hamartoma may also be associated with raised serum AFP. Therefore, keep an open mind, and an open differential list, throughout the investigative process.
  - If the tumor is seen to arise in the left lobe of the liver, the fibrolamellar variant of pediatric HCC should be considered
  - A large and clinically aggressive tumor at presentation invites the possibility of HCN-NOS
  - FDG-PET may fail to distinguish differentiated fetal HB from normal infant liver, a pitfall that persists through histologic assessment.
  - Be wary if the radiological appearance of the tumor does not match what you are seeing histologically. Again, a gross map is helpful here, which can be used as a bridge between the clinical impression and the microscopic appearance.
5. *Are you relying on AFP serology?*
  - Serum AFP levels can remain raised physiologically until 6 months of age, impacting its reliability as a tumor marker within this age group.
  - Less well-differentiated subtypes such as small cell undifferentiated HB may show normal serum AFP levels.
  - HCN-NOS typically shows highly elevated serum AFP, which may serve as further context in forming a differential list
  - AFP is undoubtedly a useful marker for tumor recurrence and progression, however the specific value must be taken within the context of clinical presentation and histology.
6. *Are you showing your cases?*
  - There is poor consensus among experts regarding a final diagnosis, in cases of mixed subtypes and those involving minor undifferentiated components.
  - The Children's Oncology Group (COG), Société Internationale D’oncologie Pédiatrique (SIOP), College of American Pathologists (CAP), United Kingdom Children's Cancer Study Group (UKCCSG), and the Canadian Association of Pathologists – Association canadienne des pathologistes (CAP-ACP) recommend establishing a process of centralized case review. An individual pathologist or center does not routinely view enough cases to develop a reliable internal resource bank, and consultation for expert opinion is consistently recommended.
  - Although consensus is not always achieved, each new case has the potential to add valuable knowledge to a slender collection of cases in the circulating literature.
7. *What about pediatric HCC?*
  - In slightly older children, pediatric HCC encroaches into the differential list. In particular, the macrotrabecular pattern of HB represents a diagnostic challenge, as the two can be morphologically similar in the narrow window of a core biopsy.
  - There is significant overlap between the immunohistochemical and genetic profile of the two entities.
  - Beta-catenin is generally less intense in a nuclear distribution in pediatric HCC when compared with HB.
  - HB is favored in the presence of glutamine synthetase and cyclin D1 positivity.
  - HB is more often associated with chromosome 1p abnormalities and trisomy of chromosomes 2, 20 and 8 than pediatric HCC.
8. *Is your immunohistochemical panel bespoke?*
  - The routine immunostains recommended to support HB diagnosis are: beta catenin, glypican 3, glutamine synthetase, and INI1.
  - INI1 is of particular importance in differentiating the prognostically poor small cell undifferentiated subtype from malignant rhabdoid tumors.
  - Based on the clinical context and radiological appearance, the panel of immunostains can be adapted to rule out common differential diagnoses, such as pediatric HCC and mesenchymal hamartoma, although currently no panel of stains can HCC from HB with absolute confidence.
  - In the context of well-differentiated fetal HB, distinction from normal liver may be achieved using immunohistochemistry, as it may look extremely similar to normal neonatal liver.

HB is a challenging diagnosis for the pathologist and her clinical colleagues, owing to its rare and heterogeneous nature. The clinical course is often non-specific until the later stages of disease, and biochemical assessment is variably fruitful. Serum AFP levels represent an important tumor marker for HB but are non-specific, complicated further by the lack of expression in the prognostically unfavorable small cell undifferentiated subtype.^5,7^ Many pitfalls may be encountered on the path from clinical presentation through to histopathological assessment, all of which must be accounted for in the formulation of a diagnosis. As cases continue to emerge and make their way into our current body of knowledge, tumor behavior will be more clearly defined. Facets of the beta-catenin signaling pathway show promise as therapeutic targets and perhaps as prognostic indicators.^10,26,29,30^ The management of HB involves radical and life altering therapy, such as chemotherapy, tumorectomy and transplantation.^1^ By identifying pitfalls as each stage of the diagnostic process, an accurate and timely diagnosis may be achieved, the importance of which cannot be understated.[Fig fig1-10668969211070178][Fig fig2-10668969211070178]
